# Effect of embryo transfer depth on IVF/ICSI outcomes: A randomized clinical trial

**DOI:** 10.18502/ijrm.v13i9.7667

**Published:** 2020-09-20

**Authors:** Robab Davar, Seyedeh Mahsa Poormoosavi, Fereshteh Mohseni, Sima Janati

**Affiliations:** ^1^Research and Clinical Center for Infertility, Yazd Reproductive Sciences Institute, Shahid Sadoughi University of Medical Sciences, Yazd, Iran.; ^2^Department of Histology, School of Medicine, Dezful University of Medical Sciences, Dezful, Iran.; ^3^Department of Obstetrics and Gynecology, School of Medicine, Dezful University of Medical Sciences, Dezful, Iran.

**Keywords:** Embryo transfer, Endometrium, Pregnancy outcomes, IVF, ICSI

## Abstract

**Background:**

Although there has been remarkable advancement in the field of assisted reproductive technology, implantation failure remains a significant issue in most infertile couples receiving these treatments. Embryo transfer is important in assisted reproductive technology and directly affects the implantation rates and pregnancy outcomes.

**Objective:**

To assess the effect of two different distance embryo transfer sites from fundal endometrial surface on the outcomes of in-vitro fertilization (IVF)/intracytoplasmic sperm injection (ICSI) cycles.

**Materials and Methods:**

A total of 180 women who were candidate for IVF/ ICSI/ embryo transfer in Yazd Research and Clinical Center for Infertility were equally assigned to two groups based on the distance between the fundal endometrial surface and catheter tip to investigate implantation, chemical and clinical pregnancy (group A: 15 ± 5 mm and group B: 25 ± 5 mm, respectively).

**Results:**

The subjects in the group B showed significantly higher implantation rate, chemical and clinical pregnancy rate compared to the group A (p = 0.03, 0.01, 0.04, respectively). The rate of ongoing pregnancy and miscarriage indicated no significant differences between groups (p = 0.21, 0.27, respectively).

**Conclusion:**

In conclusion, our study showed that the depth of embryo replacement inside the uterine cavity at a distance of 25 ± 5 mm beneath fundal endometrial surface have better effects on the pregnancy outcomes of IVF/ICSI cycles and can be considered as an important factor to improve the success of IVF cycles.

## 1. Introduction

Approximately 7% of deliveries in developed countries are performed using assisted reproductive techniques (ART) (1). Pieces of evidence suggest that 80-85% of the embryos that are replaced in the uterine cavity are miscarried and only one-fifth of the women undergoing this procedure can have an ongoing pregnancy (2). Several factors affect the rate of pregnancy following embryo transfer, including embryo number and quality (3) and uterine receptivity (4). Furthermore, several factors may influence the quality of embryo transfer, such as uterine contractions (5, 6); type of catheter (7-11); ultrasound (US) guidance; catheter-loading method; pollution of the catheter tip with blood, mucus, and bacteria; uterine cavity fluid; retained embryos; trial transfer and catheter tip placement (12-15). Embryo transfer site is considered to be a significant influential factor in the success rate of the procedure. According to the literature, if the embryo transfer depth is at or within 10 mm from the fundal endometrial surface, the pregnancy and implantation rates may decrease significantly compared to the depths of 10-20 mm (12, 16, 17). However, some studies have reported no significant differences in pregnancy and implantation rates between various absolute embryo transfer positions (e.g., 10-15, 16-21, and ≥ 21 mm) that concluded implantation rates better in 16-21 and ≥ 21 mm distance that the best place to improve the outcomes of embryo transfer has to be in the area of the mid-cavity of the uterus (18). In addition, no significant differences have been reported in the rates of pregnancy and implantation in the embryos located in the upper and lower segments of the endometrial cavity (13). Additionally, several researchers associate a higher prevalence of ectopic pregnancy after an embryo transfer close to the fundus (14). Also, another hypothesis suggests that transferring embryos to the lower portion of the uterine can prevent strong funds-contractions caused by the touching of the catheter with the uterine fundus (13).

Given the importance of embryo transfer and considering the controversies regarding the optimal location of embryo deposition, the present study aimed to assess the effects of two different distance embryo transfer sites from fundal endometrial surface on the outcomes of in-vitro fertilization/intracytoplasmic sperm injection (IVF/ICSI) cycles.

## 2. Materials and Methods

### Patients

This single-blind randomized clinical trial study was conducted on 180 women receiving IVF/ICSI at the Research and Clinical Center for Infertility, Yazd Reproductive Sciences Institute affiliated to the Shahid Sadoughi University of Medical Sciences, Yazd, Iran. The participants were aged Between 20 and 39 yr and received fresh embryo transfers.

Women with endocrine and metabolic syndromes; follicle-stimulating hormone level of > 12 and other protocols of ovarian stimulation; presence of polycystic ovarian syndrome, uterine myoma, hydrosalpinges, endometriosis (grades III, IV); history of hysteroscopic surgery due to submucosal myoma and intrauterine synechia were excluded from the study.

On the day of the embryo transfer, computer-generated random numbers were used to assign the participants into two equal groups based on the distance between the fundal endometrial surface and catheter tip, which was 15 ± 5 and 25 ± 5 mm in the group A and group B, respectively.

### Ovarian stimulation protocol

Based on the long agonist protocol for pituitary downregulation and endogenous gonadotropin depletion, the subjects in both groups received buserelin (0.5 mg; Superfact, Aventis, Frankfurt, Germany) daily via subcutaneous injection on the 21${}^{st}$ day of the cycle, following the gonadotropin administration. After menstruation, we decreased the drug dosage to 0.25 mg/day until the administration of human chorionic gonadotropin (HCG)*.* After the onset of menstruation, gonadotropin stimulation was performed using Gonal-F (Gonal-F, Serono, Aubonne, Switzerland) since day 2 of the menstruation. Based on the age and body weight of the patients, the initial dose of gonadotropin was considered to be 150-300 IU/day.

On the seventh day of ovarian stimulation, serial ultrasound monitoring was performed, and the ovarian response was assessed based on serum estradiol (E2) levels. Thereafter, the required adjustment was carried out in terms of the gonadotropin dosage. Furthermore, HCG (Pregnyl, Organon, Oss, the Netherlands) was administered at the intramuscular (IM) route with a dosage of 10,000 IU when a minimum of three follicles reached the mean diameter of ≥ 18 mm.

The oocyte retrieval was carried out in accordance with ultrasound guidelines approximately 34-36 hr after the HCG administration. In the next stage, the oocytes were transferred to pre-equilibrated IVF culture medium and incubated in CO${}_{2}$ at 37°C. Clinically appropriate IVF or ICSI were applied for oocyte insemination. After performing routine IVF/ICSI, the oocytes were transferred to culture medium containing 5% O${}_{2}$ and 6% CO${}_{2}$ for incubation at 37°C. The oocytes were also assessed in terms of fertilization 16-18 hr after the injection in order to verify the presence of pronuclei using a Nikon inverted microscope. Within 2-3 days, embryo transfer was performed in accordance with the ultrasound guidelines. Simultaneously, for the patients were administered with IM progesterone in oil (100 mg) and estradiol valerate 4 mg (Aburaihan Co., Tehran, Iran) for luteal support daily.

### Embryo transfer procedure

Fertilization and embryo quality were assessed by a skilled embryologist, and the embryos for transfer were selected according to morphologic characteristics. This way, without fragmentation, equal-sized homogenous blastomeres and cytoplasm was regarded as Grade A, embryos with ≤ 10% fragmentation, equal sized homogenous blastomeres were considered as Grade B, and embryos with ≤ 50% fragmentation, unequal sized blastomeres, and large granules as Grade C. Grades A and B embryos were defined as high- and good-quality embryos. In total, 1-3 same-quality and quantity embryos were transferred in each patient. The number of embryos to be transferred in the patients was determined based on their age and the quality of the embryos. The procedures were undertaken in the same operation room by a single gynecologist.

To perform the embryo transfer, the catheter (Frydman Classic Catheter 4.5 CCD; Laboratoire CCD, Paris, France) was loaded by the embryologist and handed to the clinician. Initially, the catheter was loaded with G1 culture medium (20 μl) (Vitrolife, Sweden). Following that, the transfer medium containing the embryo was loaded in to the catheter between air bubbles, and the transfer medium was added (up to 40 μl) for the total volume. It is notable that Mock embryo transfer was carried out in all the subjects in order to determine the depth of the uterine cavity and enrollment in IVF/ICSI. The catheter tip could be clearly viewed by the clinician based on the ultrasonography guide. The experiment was performed with the patient in the lithotomy position with full bladder. Transabdominal ultrasound was utilized to visualize the cervix, cervical canal, endometrial cavity, and uterus. Cervical exposure was performed under sterile conditions using a proper speculum to aspirate the cervical mucus from the external os using a Mucat (Laboratoire C.C.D., France).

**Figure 1:**
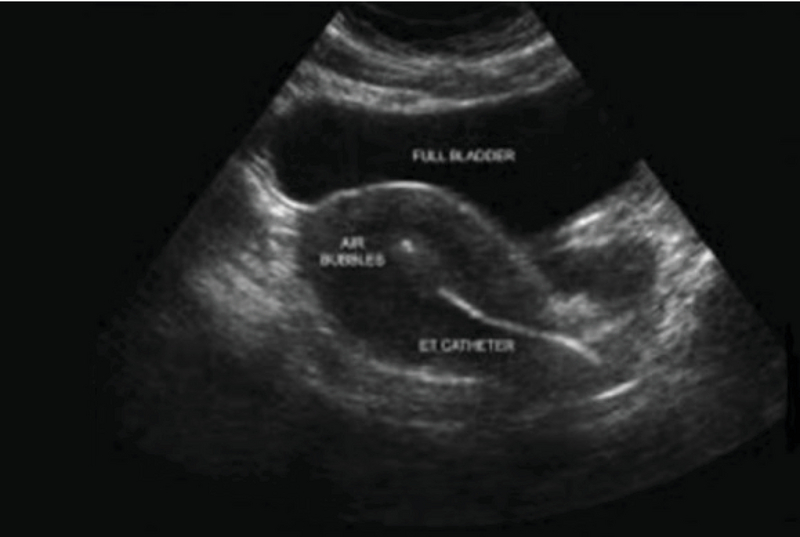
Transabdominal ultrasound viewing embryo transfer catheter and presence of air bubble at the fundus (19).

**Figure 2:**
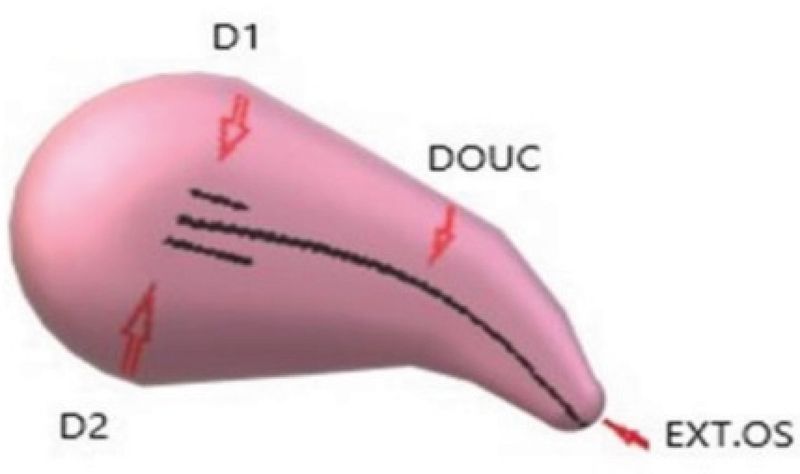
Embryo fundal distance. D1: Distance from endometrial surface and embryo deposition in group A; D2: Distance from endometrial surface and embryo deposition in group B; EXT.OS: external cervical os; DOUC: Double-outlined uterine cavity.

In the next stage, the transabdominal ultrasonography guide was used to deposit the embryos in the group A and group B at 15 ± 5 and 25 ± 5 mm below the fundus, respectively. Following that, the outer sheath of the catheter was moved toward the internal os based on the same guide. After confirming the proper position of the catheter, the embryos were loaded into the inner sheath and moved toward the endometrial cavity in order to push the embryos inside in accordance with the transabdominal ultrasonography guide and the embryos were viewed in the form of 'air bubble' (Figure 1). The distance between the air bubble and endometrial cavity fundus was measured in the freeze-frame ultrasound, and the embryos were categorized into two groups. While the embryos in the group A were transferred at 15 ± 5 mm from the fundal endometrial surface, in the group B, the distance was 25 ± 5 mm (Figure 2). Thereafter, the inner sheath was slowly withdrawn, while the outer sheath remained stable in the internal os. While the inner sheath of the catheter was closely examined by embryologists for retained embryos, the outer sheath was gradually removed in case the catheter was confirmed to be clear. The catheter tip was also examined to detect embryos or blood.

Chemical pregnancy was defined as the serum Beta HCG level > 25 IU/l within 14 days after the embryo transfer. In addition, clinical pregnancy was considered by detecting the fetal heart activity and determining the number of gestational sacs via transvaginal or abdominal ultrasonography within 2-3 wk after the confirmation of positive β-HCG. Abortion was described as the loss of pregnancy within 6-7 and < 20 wk, and ongoing pregnancy was defined as pregnancy proceeding beyond ≥ 20 wk of gestation. In the present study, the implantation rate was defined as the number of identified gestational sacs in ultrasonography per the number of the transferred embryos. Both study groups were matched in terms of the outcomes, and luteal phase support was sustained until the 10${}^{\mathrm{th}}$ week of gestation.

### Ethical consideration

Written informed consent was obtained from all participants prior to the participation. Additionally, the study protocol has been approved by the Ethics Committee of the Research and Clinical Center for Infertility, Yazd Reproductive Sciences Institute affiliated to the Shahid Sadoughi University of Medical Sciences in Yazd, Iran (Number 3772 - 16/02/2011).

### Statistical analysis

Data were analyzed using the SPSS version 20 (SPSS Inc., Chicago. IL, USA). The intergroup differences in the quantitative variables with normal distribution were assessed using student's *t* test, while Mann-Whitney U-test was applied to determine the differences in the variables with non-normal distribution. Moreover, Chi-square and Fisher's exact tests were used to compare the qualitative variables, and the obtained data were expressed as mean and standard deviation. In all the statistical analyses, the two-sided P-value < 0.05 was considered significant.

## 3. Results

**Figure 3:**
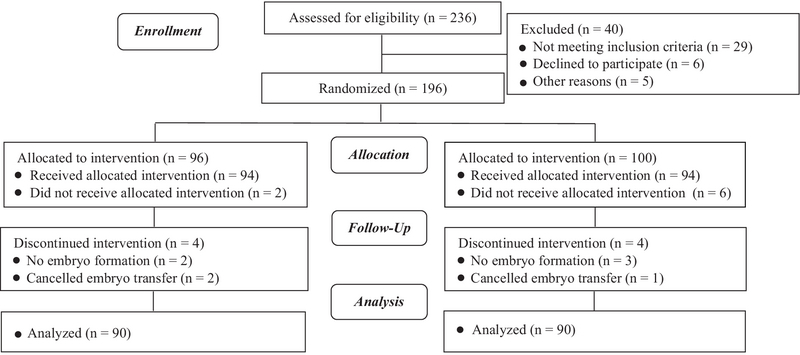
Consort flow diagram.

In total, 180 women were enrolled in the study and received embryo transfer. The subjects were divided into groups based on the distance between the fundal endometrial surface and catheter tip, which was 15± 5 and 25± 5 mm in the group A and group B, respectively. The consort statement flow diagram is presented in Figure 3.

The study groups were homogenous in terms of demographic characteristics. Furthermore, no significant differences were observed in the age, frequency of IVF/ICSI cycles, duration of infertility, number and quality of the transferred embryos, and number of the retrieved oocytes (p > 0.05) (Table I). The subjects in group B showed significantly higher implantation (17.2%), chemical pregnancy (30.4%), and clinical pregnancy (26.7%) rates compared to the group A (p < 0.05)which were estimated at 6.8%, 14.4%, and 14.4%, respectively, (p = 0.03, 0.01, 0.04, respectively). Although, no significant difference was observed in the rate of ongoing pregnancy between the two groups, this variable showed a rising trend in the group B (p = 0.21)

The obtained results indicated no significant difference in the rate of miscarriage between groups (6.7% and 2.2% respectively) (p = 0.27). Of note, no ectopic pregnancy occurred in this clinical trial, and no significant difference was observed in the cancelation rate between groups (0% and 2% respectively) (p = 0.05). Also no significant difference was observed in the rate of multifetal pregnancy between groups. (2.2% and 1.1% respectively) (p = 0.68) (Table II).

**Table 1:** Baseline and cycle characteristics of women in two groups


**Variables**		**Group A (n = 90)**	**Group B (n = 90)**	**p-value**
	Age (yr)*	29.38 ± 4.09	30.22 ± 4.5	0.25a
	Duration of infertility (yr)*	7.61 ± 4.65	7.23 ± 3.59	0.53a
	Number of retrieved oocytes *	8.41 ± 4.24	6.82 ± 3.10	0.07a
	Number of embryos transferred*	2.38 ± 0.66	2.23 ± 0.61	0.10a
Score of embryos**				
	**A**	39.60%	36.20%	
	**B**	41.20%	38.40%	
	**C**	19.20%	25.40%	0.24b
Number of IVF /ICSI cycles***				
	**IVF**	30 (33.3)	27 (30)		
	**ICSI**	60 (66.7)	63 (70)	0.74b
Kind of infertility***				
	**Primary**	84 (93.3)	84 (93.3)	
	**Secondary**	6 (6.7)	6 (6.7)	1.00b
Etiology of infertility***				
	**Male factor (%)**	61 (67.7)	58 (64.4)	
	**Tubal factor (%)**	7 (7.8)	8 (8.9)	
	**Ovarian factor (%)**	14 (15.6)	17 (18.9)	
	**Unexplained (%)**	8 (8.9)	7 (7.8)	0.71b
* Data presented as Mean ± SD. ** Data presented as percentages. *** Data presented as n (%). a independent samples *t* test b Chi-square test IVF: In vitro fertilization; ICSI: Intracytoplasmic sperm injection. P < 0.05 was considered statistically significant				

**Table 2:** ART Outcome in both groups


**Variables**	**Group A (n = 90)**	**Group B (n = 90)**	**p-value**
**Implantation rate**	6.80%	17.20%	0.03
**Chemical pregnancy rate**	13 (14.4)	30.4	0.01
**Clinical pregnancy rate**	13 (14.4)	24 (26.7)	0.04
**On-going pregnancy rate**	11 (12.2)	17 (18.8)	0.21
**Miscarriage rate**	2 (2.2)	6 (6.7)	0.27
**Multiple pregnancy rate**	2 (2.2)	1 (1.1)	0.68
Data presented as n (%). using Student *t* test, Mann-Whitney U-test, Fisher's exact test, and Chi-square test; p < 0.05 was considered statistically significant			

## 4. Discussion 

Despite the recent advancement in ART, these procedures are still associated with relatively low implantation and pregnancy rates. Therefore, the influential factors in the success rate of ART must be investigated thoroughly. As a matter of recent debate, embryo transfer may yield variable outcomes depending on the uterine site of transfer (12, 14, 19-21). The present study aimed to assess the effects of embryo transfer on the outcomes of ART. The research hypothesis was that the deposition of embryos at the mean distance of 25 ± 5 mm below the fundal endometrial surface could enhance ART outcomes.

The baseline characteristics of study population in this study were alike. The ovulation protocol and trigger were same. Moreover, the specialist reproductive endocrinologist and embryologist were same for all patients and all embryo transfers were done by the same clinician, using the same type of catheter. So, the groups examined were guarded from probable biases average of the transfer procedure. According to the obtained results, the rates of Implantation and clinical and chemical pregnancies were significantly higher in the women who received embryo transfer with the mentioned distance. According to controlled ultrasound-guided studies, the distances of 19.3 and 14.6 mm of the catheter tip from the fundal endometrial surface are associated with a significantly higher implantation rate compared to the distance of 10.2 mm. In general, the distance range of 10-20 mm has been reported to be the optimal distance from the fundus to place embryos (12, 16, 22). On the other hand, Cochrane reviews and randomized trials have reported significantly higher pregnancy rates with embryo transfer performed in accordance with ultrasound guidance, which denote that embryos should be placed in the mid-segment of the uterine cavity (23). A recent study suggests that even the smallest stimulations such as contacting the uterine fundus with the soft catheters can cause strong fundo-uterine cramps. This can lead to the movement of embryos from the superior portion of the uterine cavity into the cervix or the fallopian tubes which can result in decreasing the implantation and pregnancy rates (24).

In the current study, the embryos were placed in the uterine cavity by correct method under abdominal ultrasonographic guidance, a basic point of the study. In ultrasound-guided embryo transfer, the embryos are transferred at a specified distance from the fundal endometrial surface. It is believed by many IVF groups that a successful embryo transfer requires the endometrial fundus to not be contacted with the catheter during the replacement of the embryos (19).

Previous studies have indicated that the appropriate embryo transfer should be carried out based on the length of the uterine cavity rather than by determining a definite site (10). Several studies have shown that the deposition of embryos at the distances of > 10, > 15, and > 20 mm from the fundus could positively influence the pregnancy rate in distance of 10-15 mm (17). Also, some studies have reported the optimal site to improve the outcomes of embryo transfer in the vicinity of the mid-segment of the endometrium (18, 25). These findings are consistent with a large body of evidence in sample studies, indicating higher rates of pregnancy and implantation with the transfer of embryos into the depth of 10-20 mm of the uterine cavity (12, 16, 22). For instance, Pope and colleagues investigated the correlation between the embryo transfer site and pregnancy rate using the logistic regression model, reporting that the odds of clinical pregnancy increased by 11% with each mm of distancing of the transferred embryos from the uterine fundus (14). Similarly, Waterstone and co-workers claimed that the deposition of embryos inside the uterine cavity could positively affect the success rate of the procedure (26), which is in congruence with the findings of Naaktgeboren and others (27). Another research in this regard showed that the distances of < 7.5, 7.5-15, and 15-20 mm between the catheter tip and uterine fundus significantly increased the rates of pregnancy and implantation in distances of 15-20 mm. The researchers concluded that the depth of embryo deposition inside the uterine cavity is a significant influential factor in the success rate of ICSI (28).

In another study, Tanksale and co-authors stated that the selective deposition of embryos at a distance of 10-15 mm from the fundal endometrial surface resulted in higher pregnancy rates (29). In contrast, the other findings in this regard have reported that depositing the embryos closer to the uterine fundus could increase the rate of pregnancy (11, 30). Moreover, other studies have implied that the deposition of embryos lower into the uterine cavity could yield better outcomes in embryo transfer (9, 12, 19, 21, 26, 27, 31). Some researchers have also proposed that embryo transfer does not play a key role in implantation (22, 32). Also, according to the findings of Franco and colleagues, the rates of implantation and pregnancy are not influenced by the deposition of the embryo in the upper or lower segments of the uterus (13).

Moreover, Tiras and co-authors reported that the unintentional deposition of embryos at a distance of 10 mm from the uterine fundus may not have an impact on the rate of pregnancy. However, embryo deposition at distances > 10 mm from the fundus at any site within the uterine cavity does not adversely affect the pregnancy rate, while the distances between > 10 mm and < 20 mm seem optimal for embryo transfer, resulting in higher pregnancy rates (22). In the current research, the rate of spontaneous abortion was consistent with the previous findings in this regard, which denoted no correlations between the site of embryo deposition in the endometrial cavity and abortion rate (13, 22, 25). Similarly, Tiras and colleagues reported that the rate of miscarriage is not affected by the site of embryo transfer with established pregnancy (22). In contrast, no significant trend was reported in the study by Oliviere regarding the increased rate of abortion when the embryos released ≤ 40% of the endometrial cavity length (18). In addition, another study indicated that the rate of spontaneous miscarriage was significantly higher in the subjects undergoing clinical touch embryo transfer compared to those receiving ultrasound-guided embryo replacement (5, 12).

No ectopic pregnancy occurred in the current research, while other studies in this regard have reported that the risk of ectopic pregnancy may increase with the transfer of embryos close to the uterine fundus (8, 13, 21, 33, 34).

## 5. Conclusion 

According to the results, the rates of implantation, chemical and clinical may be influenced by the site of embryo transfer. Moreover, favorable outcomes were achieved with the catheter tip at the distance of 25 ± 5 mm beneath the fundal endometrial surface and it can be considered as an important factor to improve the success of IVF cycles.

##  Conflicts of Interest

The authors declare that there is no conflict of interest.

## References

[1] Eftekhar M, Janati S, Rahsepar M, Aflatoonian A. Effect of oocyte activation with calcium ionophore on ICSI outcomes in teratospermia: A randomized clinical trial. *Iran J Reprod Med* 2013; 11: 875–882. PMC3941389 24639711

[2] De Neubourg D, Gerris J, Mangelschots K, Van Royen E, Vercruyssen M, Elseviers M. Single top quality embryo transfer as a model for prediction of early pregnancy outcome. *Hum Reprod* 2004; 19: 1476–1479. 10.1093/humrep/deh283 15117893

[3] Strandell A, Bergh C, Lundin K. Selection of patients suitable for one-embryo transfer may reduce the rate of multiple births by half without impairment of overall birth rates. *Hum Reprod* 2000; 15: 2520–2525. 10.1093/humrep/15.12.2520 11098020

[4] Schoolcraft WB, Surrey ES, Gardner DK. Embryo transfer: techniques and variables affecting success. *Fertil Steril* 2001; 76: 863–870. 10.1016/s0015-0282(01)02731-5 11704102

[5] Coroleu B, Carreras O, Veiga A, Martell A, Martinez F, Belil I, et al. Embryo transfer under ultrasound guidance improves pregnancy rates after in-vitro fertilization. *Hum Reprod* 2000; 15: 616–620. 10.1093/humrep/15.3.616 10686207

[6] Matorras R, Urquijo E, Mendoza R, Corcostegui B, Exposito A, Rodriguez-Escudero FJ. Ultrasound-guided embryo transfer improves pregnancy rates and increases the frequency of easy transfers. *Hum Reprod* 2002; 17: 1762–1766. 10.1093/humrep/17.7.1762 12093836

[7] Aichberger L, Boldizsar A, Herczeg C, Obermair A, Plockinger B, Strohmer H, et al. [Vaginal ultrasonographic observation of uterine contractions in embryo transfer and its relevance to treatment success]. *Geburtshilfe Frauenheilk* 1991; 51: 27–30. (in German) 10.1055/s-2008-1026327 2026297

[8] Baba K, Ishihara O, Hayashi N, Saitoh M, Taya J, Kinoshita K. Where does the embryo implant after embryo transfer in humans? *Fertil Steril* 2000; 73: 123–125. 10.1016/s0015-0282(99)00454-9 10632425

[9] Frankfurter D, Silva CP, Mota F, Trimarchi JB, Keefe DL. The transfer point is a novel measure of embryo placement. *Fertil Steril* 2003; 79: 1416–1421. 10.1016/s0015-0282(03)00263-2 12798891

[10] Frankfurter D, Trimarchi JB, Silva CP, Keefe DL. Middle to lower uterine segment embryo transfer improves implantation and pregnancy rates compared with fundal embryo transfer. *Fertil Steril* 2004; 81: 1273–1277. 10.1016/j.fertnstert.2003.11.026 15136089

[11] Krampl E, Zegermacher G, Eichler C, Obruca A, Strohmer H, Feichtinger W. Air in the uterine cavity after embryo transfer. *Fertil Steril* 1995; 63: 366–370. 10.1016/s0015-0282(16)57370-1 7843445

[12] Coroleu B, Barri PN, Carreras O, Martinez F, Parriego M, Hereter L, et al. The influence of the depth of embryo replacement into the uterine cavity on implantation rates after IVF: a controlled, ultrasound-guided study. *Hum Reprod* 2002; 17: 341–346. 10.1093/humrep/17.2.341 11821275

[13] Franco Jr JG, Martins AM, Baruffi RL, Mauri AL, Petersen CG, Felipe V, et al. Best site for embryo transfer: the upper or lower half of endometrial cavity? *Hum Reprod* 2004; 19: 1785–1790. 10.1093/humrep/deh308 15218006

[14] Pope CS, Cook EK, Arny M, Novak A, Grow DR. Influence of embryo transfer depth on in vitro fertilization and embryo transfer outcomes. *Fertil Steril* 2004; 81: 51–58. 10.1016/j.fertnstert.2003.05.030 14711544

[15] van Weering HG, Schats R, McDonnell J, Vink JM, Vermeiden JP, Hompes PG. The impact of the embryo transfer catheter on the pregnancy rate in IVF. *Hum Reprod* 2002; 17: 666–670. 10.1093/humrep/17.3.666 11870120

[16] Abou-Setta AM. What is the best site for embryo deposition? A systematic review and meta-analysis using direct and adjusted indirect comparisons. *Reprod Biomed Online* 2007; 14: 611–619. 10.1016/s1472-6483(10)61054-1 17509204

[17] Pacchiarotti A, Mohamed MA, Micara G, Tranquilli D, Linari A, Espinola SM, et al. The impact of the depth of embryo replacement on IVF outcome. *J Assist Reprod Genet* 2007; 24: 189–193. 10.1007/s10815-007-9110-4 PMC3455059 17342426

[18] Oliveira JB, Martins AM, Baruffi RL, Mauri AL, Petersen CG, Felipe V, et al. Increased implantation and pregnancy rates obtained by placing the tip of the transfer catheter in the central area of the endometrial cavity. *Reprod Biomed Online* 2004; 9: 435–441. 10.1016/s1472-6483(10)61280-1 15511345

[19] Woolcott R, Stanger J. Potentially important variables identified by transvaginal ultrasound-guided embryo transfer. *Hum Reprod* 1997; 12: 963–966. 10.1093/humrep/12.5.963 9194648

[20] Lesny P, Killick SR, Tetlow RL, Robinson J, Maguiness SD. Embryo transfer–can we learn anything new from the observation of junctional zone contractions? *Hum Reprod* 1998; 13: 1540–1546. 10.1093/humrep/13.6.1540 9688388

[21] Nazari A, Askari HA, Check JH, O'Shaughnessy A. Embryo transfer technique as a cause of ectopic pregnancy in in vitro fertilization. *Fertil Steril* 1993; 60: 919–921. 8224280

[22] Tiras B, Polat M, Korucuoglu U, Zeyneloglu HB, Yarali H. Impact of embryo replacement depth on in vitro fertilization and embryo transfer outcomes. *Fertil Steril* 2010; 94: 1341–1345. 10.1016/j.fertnstert.2009.07.1666 20044085

[23] Sallam HN. Embryo transfer: factors involved in optimizing the success. *Curr Opin Obstet Gynecol* 2005; 17: 289–298. 10.1097/01.gco.0000169107.08000.dd 15870564

[24] Ivanovski M, Popovska S. The impact of the depth of embryo replacement into the uterine cavity under transabdominal ultrasound guidance on in vitro fertilization and embryo transfer outcome. *Maced J Med Sci* 2013; 6: 376–382.

[25] Cavagna M, Contart P, Petersen CG, Mauri AL, Martins AM, Baruffi RL, et al. Implantation sites after embryo transfer into the central area of the uterine cavity. *Reprod Biomed Online* 2006; 13: 541–546. 10.1016/s1472-6483(10)60642-6 17007675

[26] Waterstone J, Curson R, Parsons J. Embryo transfer to low uterine cavity. *Lancet* 1991; 337: 1413. 10.1016/0140-6736(91)93094-p 1674783

[27] Naaktgeboren N, Dieben S, Heijnsbroek I, Verburg H, Van der Westerlaken L. Embryo transfer, easier said than done. *Fertility and Sterility* 1998; 70 (Suppl.): S352.

[28] Salam Mohamed MA. The influence of the depth of embryo transfer into the uterine cavity on implantation rate. *Middle East Fertil Soc J* 2010; 15: 174–178.

[29] Tanksale SJ NP, Nadkarni AA, Singh P. Where is the best site for embryo transfer? A study of relation of embryo-fundal distance with pregnancy rate in ICSI-ET cycle. *Int J Reprod Contracept Obstet Gynecol *2016; 5: 2661–2665.

[30] Meldrum DR, Chetkowski R, Steingold KA, de Ziegler D, Cedars MI, Hamilton M. Evolution of a highly successful in vitro fertilization-embryo transfer program. *Fertil Steril* 1987; 48: 86–93. 10.1016/s0015-0282(16)59295-4 2954865

[31] van de Pas MM, Weima S, Looman CW, Broekmans FJ. The use of fixed distance embryo transfer after IVF/ICSI equalizes the success rates among physicians. *Hum Reprod* 2003; 18: 774–780. 10.1093/humrep/deg175 12660270

[32] Rosenlund B, Sjoblom P, Hillensjo T. Pregnancy outcome related to the site of embryo deposition in the uterus. *J Aassist Reprod Genet* 1996; 13: 511–513. 10.1007/BF02066534 8835682

[33] Egbase PE, Al-Sharhan M, Grudzinskas JG. Influence of position and length of uterus on implantation and clinical pregnancy rates in IVF and embryo transfer treatment cycles. *Hum Reprod *2000; 15: 1943–1946. 10.1093/humrep/15.9.1943 10966991

[34] Yovich JL, Turner SR, Murphy AJ. Embryo transfer technique as a cause of ectopic pregnancies in in vitro fertilization. *Fertil Steril* 1985; 44: 318–321. 10.1016/s0015-0282(16)48854-0 4029420

